# Australian population norms for health-related quality of life measured using the EQ-5D–5L, and relationships with sociodemographic characteristics

**DOI:** 10.1007/s11136-023-03558-z

**Published:** 2023-12-12

**Authors:** Lisa Redwood, David Currow, Slavica Kochovska, Susan J. Thomas

**Affiliations:** 1 Graduate School of Medicine, Faculty of Science, Medicine and Health, University of Wollongong, Wollongong, NSW Australia; 2 Mental Illness in Nowra District: Goals and Prevention (MIND the GaP), University of Wollongong, Wollongong, NSW Australia; 3 Faculty of Science, Medicine and Health, University of Wollongong, Wollongong, Australia

**Keywords:** EQ-5D-5L, EQ VAS, Population norms, Reference values, Health-related quality of life

## Abstract

**Background:**

Measuring health related quality-of-life (HRQoL) of the general population is essential to establish a reference for health outcome evaluations. This study sought to establish EQ-5D-5L population norms in Australia and to investigate the heterogeneity of HRQoL between sociodemographic variables.

**Methods:**

A cross-sectional study comprising of a representative sample of Australia’s general population (n = 9958) aged 18 or older. Recruitment quotas were set for the Australian census population by age, sex, state/territory of residence and rurality. Participants were recruited by Qualtrics through its database of over 800,000 registered panel members and asked to value their own state of health using the EQ-5D-5L domains and the EuroQol-Visual Analogue Scale (EQ-VAS). An Australian value set developed using Discreet Choice Experiment was used to calculate utility scores.

**Results:**

The estimated mean EQ-5D-5L index for Australia’s general population was 0.86 (standard deviation [SD] 0.19), and the EQ-VAS score was estimated as 73.2 (SD 21.7). 23.9% of the study population reported being in the best health state (11,111). Younger people, current smokers, people who are unemployed and people with more financial stress reported a lower EQ-5D-5L index score (p < 0.001). Residents in the major cities, inner regional and outer regional Australia reported higher health utility scores than those residing in remote and very remote Australia.

**Conclusions:**

This is the first Australian study to apply the EQ-5D-5L in a nationally representative sample. The EQ-5D-5L Australian population norms obtained can be used as reference scores for future population health evaluations and comparisons. The findings facilitate a national reference for clinical, economic, and policy decision-making processes and provide a fuller understanding of the Australian population’s HRQoL.

**Supplementary Information:**

The online version contains supplementary material available at 10.1007/s11136-023-03558-z.

## Introduction

In 1946 the World Health Organization defined health as “a state of complete physical, mental, and social well-being and not merely the absence of disease or infirmity” [1]. Although this definition is one of the most widely known, it has been debated and said to lack operational value [2]. Subsequently, several self-reported Health-Related Quality of Life (HRQoL) instruments have been developed that include the generally agreed upon aspects of HRQoL: physical, mental, and social well-being [3]. HRQoL is commonly used for economic evaluations, research and health interventions and policies; and for monitoring the health status of the general population [4, 5]. A frequently used measure of economic evaluations is the quality adjusted life year (QALY) [6]. QALY is derived from a combination of HRQoL and length of life into a single index summary measure [6]. QALY has also been used to quantify health outcomes for cost-utility analyses which are broadly used to guide health care resource allocation [7]. HRQoL measures are often adopted for use in research in clinical and epidemiological studies to assess the impacts associated with health conditions on individuals’ well-being, as well as outcomes of healthcare interventions. National HRQoL surveillance can assess how a population is changing over time and identify unmet population health needs [8]. Therefore, identifying national population norms for HRQoL is an essential element for the development and evaluation of health care services.

The EQ-5D was developed in 1990 and is the world’s most widely applied HRQoL and patient-reported outcomes measure [6, 9]. The EQ-5D contains five dimensions of health: mobility, self‐care, usual activities, pain/discomfort, and anxiety/depression [9]. The original three level version (EQ-5D-3L) contains three responses to the five dimensions/questions including: no problem; some problems; and extreme problems [9]. A five-level version (EQ-5D-5L) was released in 2009 and contains five levels of responses to the five domains: no problems; slight problems; moderate problems; severe problems; and extreme problems [9]. The EQ-5D-5L has demonstrated reduced ceiling effects and improved the sensitivity of the 3L version for detecting clinically important differences in HRQoL [10–12]. Therefore, the 5L version is replacing the 3L version as the preferred measure in HRQoL population-wide studies and health economic evaluations [9].

The EQ-5D-5L is a preference-based measure widely used in cost-utility analysis. The 5L version has been validated and utilised across multiple diseases, conditions and locations. More recently, the EQ-5D-5L has been used in routine outcomes measurement in hospital settings to assess the performance and productivity of healthcare systems [15]. The utility values can easily be converted into QALY and used to conduct cost–utility analysis in economic evaluations. To achieve this, a 5L value set for the country is needed. In Australia, a 5L value set using the standardised valuation study protocol is not available, however an alternative value set using the discrete choice experiment (DCE) method was developed in 2023 [13]. Population norms for HRQoL play an important role as a reference group to facilitate comparisons among people with specific conditions with data on the average person of a similar age and/or sex within the same community [14]. They can also be compared against to assess the incremental effectiveness of interventions in economic evaluations and can be used to support policy makers identify gaps, and detect health priorities to which to allocate resources to reduce inequalities [6, 15, 16].

The EQ-5D-5L population norms and sociodemographic characteristics have been reported and validated in many countries such as Germany [17], Italy [18, 19], Japan [20], Poland [21], Spain [22], Uruguay [23], Vietnam [24] and the United Kingdom (UK) [11]. Although population norms for EQ-5D-5L have been developed for South Australia [25], none have been created for the general Australian population. Therefore, this study aimed to estimate the population norms for the EQ-5D-5L for Australia using a large nationally representative sample and the DCE based value set for Australia; and to investigate the heterogeneity of HRQoL between sociodemographic variables using the EQ-5D-5L utility and the EuroQol-Visual Analogue Scale (EQ-VAS) [26].

## Methods

### Sample size

The current cross-sectional study was conducted with a demographically representative Australian sample of adults aged 18 years or older from 12 July 2021 to 2 August 2021. This study is a part of a larger study of breathlessness in the Australian population [25]. The original sample size was calculated based on the prevalence rate of moderate to severe breathlessness in Australia (estimated to be between 1.0 and 2.6%).

### Recruitment methods

Participants were invited to complete an online survey using the Qualtrics platform (Utah, USA) through its database of over 800,000 registered panel members. Recruitment quotas were set for combinations of all four demographic parameters based on the Australian 2016 census population by age, sex, state/territory of residence and rurality [27].

The survey was piloted with members of the Improving Palliative, Aged and Chronic Care through Clinical Research and Translation (IMPACCT) Consumer Advisory Group (University of Technology Sydney) and 110 Qualtrics panellists before general recruitment. Panel members provided initial informed consent when joining Qualtrics’ panel and then, subsequently, chose and agreed to participate in this survey. The survey took between 16 and 20 min to complete. This was part of a larger study that looked at the reporting of breathlessness as a symptom in clinical consultations in Australia [28].

### Measures

#### Sociodemographic characteristics

Sociodemographic characteristics collected in the survey included age, sex, state, postcode (used to code remoteness area) [29], height and weight (used to calculate Body Mass Index (BMI)), smoking status, employment status and financial stress. Age was categorised into seven categories: 18 to 24 years, 25 to 34 years, 35 to 44 years, 45 to 54 years, 55 to 64 years, 65 to 74 years, and 75 years or older to align with other EQ-5D-5L studies. Missing data were excluded from the analyses.

#### EQ-5D-5L

EQ-5D-5L was used to assess HRQoL [10]. The EQ-5D-5L consists of five questions (dimensions) with five response levels: no problems (Level 1); slight; moderate; severe; and extreme problems (Level 5). The combination of the level from each dimension created a health score, which ranged from 11,111 (full health) to 55,555 (worst health) [10]. EQ-5D-5L health states are converted into a single index ‘utility’ score using a scoring algorithm based on public preferences. The utility score is measured on a scale from ‘0’ to ‘1’ where ‘0’ represents being dead and ‘1’ being in full health. Negative scores are possible, indicating a health state worse than death. A DCE based value set for Australia was used to calculate utility scores as a value set using the EQ-VT standardised protocol for Australia is not yet available[26, 30]. The EQ-5D-5L also contains the EQ-VAS, which is a single rating of self-perceived health and is scored on a scale ranging from 0 (worst imaginable health state) to 100 (best imaginable health state) [31].

A sensitivity analysis was conducted in the supplementary material to compare the impact of the Australian value set developed using the DCE duration valuation protocol and the US [1] and Italian [2] value sets, developed using the EQ-VT v2.1 protocol.

#### Data analysis

Descriptive statistics were used to compare the study sample with the Australian Census data 2021 [32], and to show the frequency of the response to the five domains by age category. To improve the accuracy of the results the data were weighted for age, sex, rurality and state of residence based on the 2021 Australian Census data.

As the EQ-5D-5L utility scores were non-normally distributed, non-parametric tests were used. The Mann–Whitney U test was used to compare the utility and EQ-VAS scores by sex. The Kruskal–Wallis H test was used to compare the utility and EQ-VAS scores by the categorical variables, including age category, state, remoteness area, BMI category, smoking status, employment status and financial stress. To account for multiple testing, the alpha level for the Kruskal–Wallis H test was adjusted to 0.00714 (0.05/7).

The Generalized Linear Model (GLM) was employed to explore the association between the sociodemographic variables and utility scores as well as EQ-VAS scores. This model can control skewness and heteroscedasticity. To account for overdispersion, a Quasipoisson distribution with a log link was utilised. This method requires non-negative values, therefore the disutility value (disutility = 1-utility value) was entered as the dependent variable [24]. Based on recently reported EQ-5D-5L population norms, it was hypothesised that older people and females were expected to have higher disutility scores and were entered first in the GLM (Model 1) [19, 33, 34]. Analyses were conducted using R studio and IBM SPSS Statistics 28.0 [35, 36].

#### Ethics

A participant information sheet with study details was provided before obtaining informed consent for this survey. This manuscript was written in accordance with the STROBE checklist [37]. Approval was obtained from the human research ethics committee (University of Technology Sydney; UTS HREC ETH20-5114).

## Results

This study recruited 10,034 participants. There were 76 records that contained a missing key variable. There were 9,958 participants that were included in the analyses. The sample was similar to the Australian general population (Table 1). Compared to the general Australian population, the study participants had a higher mean age (39.8 vs. 47.8 years, respectively) and more participants that resided in major cities (71.7% vs. 78.4%, respectively). There were also no participants recruited from the Northern Territory, which accounts for 0.9% of the Australian population. There were no missing data for the EQ-5D-5L health states nor the EQ-VAS.

**Table 1:** Sociodemographic characteristics of the Australian sample

	Australian census data (2021)^a^	Total	Male	Female
	n = 25,422,788	n = 9958	n = 4855	n = 5103
Age, mean (SD)	39.8	47.8 (18.3)	47.4 (18.1)	48.1 (18.4)
Age category, years, %				
18–24	10.8	10.8	5.5	5.3
25–34	17.4	18.2	9.0	9.2
35–44	16.8	17.5	8.6	8.9
45–54	15.6	16.3	8.0	8.3
55–64	14.5	15.1	7.4	7.8
65–74	11.8	12.4	6.0	6.4
75 +	9.2	9.7	4.3	5.4
State, %				
New South Wales	31.8	32.1	15.6	16.4
Victoria	25.6	25.9	12.6	13.3
Australian Capital Territory	1.8	1.8	0.9	0.9
Tasmania	2.2	2.3	1.1	1.2
Queensland	20.3	20.3	9.9	10.4
Northern Territory	0.9	0.0	0.0	0.0
South Australia	7.0	7.2	3.5	3.7
Western Australia	10.5	10.4	5.1	5.3
Remoteness area, %				
Major cities	71.7	78.4	39.1	39.2
Inner regional	18.2	16.0	7.0	9.1
Outer regional	8.2	5.2	2.2	3.0
Remote	1.2	0.3	0.1	0.2
Very remote	0.8	0.1	0.0	0.0
BMI category, %				
Underweight	N/A	4.3	1.8	2.5
Healthy weight		38.6	18.3	20.2
Overweight		31.4	18.7	12.8
Obese		25.7	11.2	14.5
Smoking, %				
I am a current smoker	N/A	23.2	13.6	9.6
I am a former smoker		28.2	14.5	13.8
I have never smoked		48.6	20.7	27.9
Employment, %				
Employed full time	N/A	37.0	23.7	13.3
Employed part-time		14.2	5.1	9.1
Employed casual		5.5	1.8	3.7
Self-employed		5.2	2.9	2.3
Retired		25.7	12.1	13.5
Unemployed		11.2	4.2	7.0
On paid leave		0.6	0.1	0.5
On unpaid leave		0.6	0.1	0.5
Financial stress				
I/We spend more money than I/we get	N/A	10.7	5.8	4.9
I/We have just enough money to get me/us through to the next pay day		23.0	10.5	12.5
There is some money let over each week but I/we just spend it		8.2	4.4	3.8
I/We can save a bit every now and then		39.9	18.7	21.2
I/We can save a lot		18.2	9.6	8.7

*SD *standard deviation

^a^Data are based on results of the 2021 Australian Census [1], where available; N/A indicates that a directly comparable question was not included in the 2021 Census

The frequencies of item responses for each EQ-5D-5L dimension, by age category, are presented in Table 2. A substantial proportion of respondents reported a problem on at least one of the five dimensions (76.1%). The most prevalent problems were reported for pain and discomfort (61.4%) followed by anxiety and depression (55.1%). In general, problems with mobility and pain/discomfort increased as age increased. In contrast, problems with anxiety and depression were higher in younger people and tended to decrease with age. This finding was robust when outliers were removed.

**Table 2:** Frequencies of item responses in each EQ-5D-5L dimension by age and gender (%)

Dimension	Age category, years							
	Total n = 9958	18–24 n = 1075	25–34 n = 1809	35–44 n = 1743	45–54 n = 1624	55–64 n = 1507	65–74 n = 1234	75 + n = 966
Mobility								
No problems	66.7	71.3	71.5	71.7	69.5	65.1	60.6	49.1
Slight	18.8	15.4	15.3	16.9	19.1	19.9	23.1	25.1
Moderate	10.0	9.4	9.4	8.8	8.1	9.1	11.2	17.1
Severe	3.5	3.5	3.1	1.8	2.0	4.7	4.4	6.4
Extreme	1.0	0.4	0.6	0.7	1.3	1.1	0.7	2.3
Self-care								
No problems	80.4	73.7	77.5	80.8	81.4	82.1	87.3	79.4
Slight	12.0	14.0	11.3	10.9	12.8	12.4	9.4	14.3
Moderate	5.6	9.4	7.5	6.6	3.5	3.9	2.6	5.8
Severe	1.4	2.0	3.1	1.4	1.0	1.1	0.5	0.5
Extreme	0.6	0.9	0.6	0.4	1.2	0.5	0.3	0.0
Usual activities								
No problems	63.4	60.6	62.0	67.5	64.3	67.1	65.0	52.5
Slight	22.2	21.9	22.9	20.6	22.0	18.5	22.9	29.3
Moderate	10.5	12.8	10.7	8.7	9.4	10.1	9.4	14.5
Severe	2.9	4.0	3.0	2.6	2.6	3.5	2.2	2.2
Extreme	1.0	0.8	1.4	0.6	1.7	0.7	0.5	1.6
Pain/discomfort								
No pain	38.6	45.6	44.5	45.5	38.5	35.2	29.3	24.2
Slight	37.1	33.3	33.7	34.7	36.1	37.0	43.8	45.4
Moderate	16.7	13.9	13.3	14.2	16.2	19.4	20.3	23.3
Severe	5.8	5.7	7.0	4.5	6.0	6.1	6.1	4.5
Extreme	1.8	1.5	1.5	1.1	3.2	2.4	0.6	2.5
Anxiety/depression								
No problems	44.9	27.7	33.2	38.8	40.5	55.3	63.4	64.5
Slight	27.5	24.4	30.7	32.3	30.3	24.5	22.6	22.9
Moderate	16.7	24.5	21.6	17.4	17.3	13.2	10.2	10.3
Severe	7.1	15.2	10.2	6.8	6.8	4.8	2.5	2.4
Extreme	3.8	8.2	4.3	4.8	5.2	2.2	1.3	0.0

Frequencies have been weighted by age, sex and state based on the 2021 Australian census, excluding Northern Territory

The mean utility score for the Australian general population was 0.86 (SD 0.19; 95% CI 0.85, 0.86) (Table 3) and the mean EQ-VAS score was 73.2 (SD 21.7; 95% CI 72.6, 73.4) (Table 4). Ten out of the 3,125 possible health states accounted for the over half of the sample (53.4%) (Table 5). Individuals rating themselves as ‘11,111’, assigned a mean score of 85.8 to their health state on the EQ-VAS.

**Table 3:** Mean EQ-5D-5L utility scores by sociodemographic characteristics

Variable	Utility scores								
	Total			Male			Female		
	n	Mean	SD	n	Mean	SD	n	Mean	SD
Total	9958	0.86	0.19	4855	0.86	0.20	5103	0.85	0.18
				Z = −7.0, p < 0.001^a^					
Age category, years									
18–24	1075	0.82	0.20	549	0.83	0.21	526	0.82	0.19
25–34	1809	0.84	0.21	894	0.83	0.24	915	0.85	0.18
35–44	1743	0.87	0.18	858	0.88	0.18	885	0.86	0.18
45–54	1624	0.85	0.21	795	0.86	0.22	829	0.84	0.20
55–64	1507	0.86	0.20	734	0.85	0.22	774	0.87	0.18
65–74	1234	0.88	0.17	596	0.89	0.16	639	0.87	0.17
75+	966	0.86	0.18	430	0.90	0.15	535	0.83	0.19
Total	9958	X^2^ = 42.5		p < 0.001^b^					
State									
New South Wales	3193	0.86	0.19	1558	0.85	0.22	1635	0.87	0.17
Victoria	2583	0.85	0.20	1256	0.85	0.21	1327	0.84	0.19
Australian Capital Territory	180	0.89	0.16	88	0.89	0.15	92	0.89	0.17
Tasmania	225	0.85	0.19	109	0.86	0.17	116	0.83	0.20
Queensland	2020	0.85	0.20	982	0.86	0.20	1037	0.83	0.19
Northern Territory	0			0			0		
South Australia	716	0.84	0.20	349	0.87	0.17	367	0.82	0.22
Western Australia	1040	0.88	0.16	512	0.88	0.17	528	0.87	0.16
Total	9958	X^2^ = 48.8		p < 0.001^b^					
Remoteness area									
Major cities	7725	0.86	0.19	3856	0.87	0.20	3869	0.86	0.17
Inner regional	1582	0.82	0.22	688	0.83	0.22	894	0.82	0.21
Outer regional	516	0.83	0.21	217	0.83	0.22	298	0.83	0.20
Remote	30	0.80	0.26	12	0.85	0.25	19	0.78	0.26
Very remote	6	0.73	0.31	3	0.72	0.18	3	0.74	0.44
Total	9859	X^2^ = 88.4		p < 0.001^b^					
BMI category									
Underweight	370	0.85	0.20	153	0.86	0.20	217	0.85	0.20
Healthy weight range	3283	0.88	0.18	1561	0.87	0.20	1722	0.88	0.16
Overweight	2675	0.88	0.17	1588	0.89	0.17	1087	0.87	0.16
Obese	2186	0.80	0.22	950	0.81	0.23	1235	0.80	0.21
Total	8514	X^2^ = 384.8		p < 0.001^b^					
Smoking status									
I am a current smoker	2282	0.80	0.24	1338	0.80	0.25	944	0.81	0.22
I am a former smoker	2777	0.84	0.19	1423	0.86	0.19	1353	0.83	0.18
I have never smoked	4781	0.89	0.17	2039	0.90	0.17	2742	0.88	0.17
Total	9841	X^2^ = 354.9		p < 0.001^b^					
Employment status									
Employed full time	3436	0.88	0.19	2205	0.87	0.21	1231	0.89	0.15
Employed part-time	1324	0.87	0.17	477	0.86	0.19	847	0.87	0.16
Employed casual	509	0.87	0.16	165	0.89	0.15	344	0.86	0.16
Self-employed	488	0.87	0.19	272	0.86	0.22	216	0.88	0.16
Retired	2387	0.86	0.19	1129	0.87	0.19	1258	0.84	0.19
Unemployed	1040	0.81	0.21	392	0.82	0.21	648	0.81	0.21
On paid leave	57	0.87	0.16	11	0.80	0.21	47	0.89	0.15
On unpaid leave	51	0.81	0.23	7	0.80	0.25	44	0.81	0.23
Total	9293	X^2^ = 188.7		p < 0.001^b^					
Financial stress									
I/We spend more money than I/we get	1000	0.75	0.27	543	0.73	0.30	456	0.77	0.24
I/We have just enough money to get me/us through to the next pay day	2157	0.81	0.22	987	0.81	0.23	1169	0.80	0.21
There is some money let over each week but I/we just spend it	769	0.85	0.18	416	0.84	0.19	353	0.85	0.18
I/We can save a bit every now and then	3744	0.88	0.16	1752	0.89	0.17	1992	0.88	0.16
I/We can save a lot	1708	0.91	0.13	896	0.93	0.12	812	0.90	0.14
Total	9377	X^2^ = 664.5		p < 0.001^b^					

Means and standard deviations have been weighted by age, sex and state based on the 2021 Australian census, excluding the Northern Territory

*SD* standard deviation, *BMI* Body Mass Index

^a^Mann–Whitney U test

^b^Kruskal–Wallis H test

**Table 4:** Mean EQ-VAS scores by age and gender

Variable all	Total			Male			Female		
	n	mean	SD	n	mean	SD	n	mean	SD
	9958	73.2	21.7	4855	73.8	22.2	5103	72.8	21.3
				Z = − 2.0 p < 0.05^a^					
Age category, years									
18–24	1075	72.3	22.2	549	73.8	22.5	526	70.7	21.8
25–34	1809	73.0	21.6	894	73.5	22.1	915	72.5	21.1
35–44	1743	72.2	23.2	858	73.4	23.6	885	71.1	22.8
45–54	1624	71.3	22.9	795	70.6	24.0	829	71.9	21.8
55–64	1507	74.0	21.1	734	71.9	21.9	774	76.0	20.1
65–74	1234	76.1	19.3	596	76.8	19.1	639	75.5	19.5
75 +	966	74.1	20.2	430	77.0	19.0	535	71.7	20.9
Total	9958	X^2^ = 38.4, p < 0.01^b^							

Means and standard deviations have been weighted by age, sex and state based on the 2021 Australian census, excluding Northern Territory

*SD* standard deviation

^a^Mann–Whitney U test

^b^Kruskal–Wallis H test

**Table 5:** Most frequently reported EQ-5D-5L health states with mean utility scores and EQ-VAS values

Health score	n	%	Mean utility	Mean EQ-VAS	(SD)
11111	2314	23.9	1.00	85.8	15.7
11112	741	7.3	0.97	81.4	15.3
11113	272	2.3	0.93	74.7	17.6
11114	61	0.5	0.76	66.7	23.0
11115	41	0.4	0.76	61.9	26.1
11121	792	8.2	0.96	83.6	11.9
11122	716	7.3	0.92	78.2	14.9
11123	276	2.6	0.89	71.9	17.1
11124	88	0.7	0.72	69.4	17.7
11125	31	0.2	0.72	56.8	23.0
11131	68	0.7	0.92	76.2	21.1
11132	53	0.6	0.89	77.9	18.3
11133	62	0.6	0.85	64.9	17.6
11134	25	0.3	0.68	58.7	24.9
11135	11	0.1	0.68	64.0	20.6
11141	9	0.1	0.72	74.2	17.3
11142	5	0.1	0.69	45.2	22.3
11143	3	0.0	0.66	58.8	1.2
11144	6	0.1	0.49	35.3	24.7

Data have been weighted by age, sex and state based on the 2021 Australian census, excluding the Northern Territory

*EQ-VAS* EuroQol-Visual Analogue Scale, *SD* standard deviation

The mean EQ-5D-5L utility scores by sociodemographic variables and mean EQ-VAS scores by age and sex are summarised in Tables 3 and 4 respectively. Overall, males had higher mean utility scores than females (0.86 (SD 0.20) versus 0.85 (SD 0.18); Z = − 7.0, p < 0.001). Lower utility scores were reported in younger age categories (X^2^ = 42.5, p < 0.001), people residing in more regional and remote Australia (X^2^ = 88.4, p < 0.001) and people experiencing financial stress (X^2^ = 664.5, p < 0.001). There were also statistically significant differences in EQ-VAS scores for age categories and sex (Table 4).

In the Quasipoisson models, advancing age was significantly associated with a positive impact on health status and disutility was higher for those who were self-employed, retired, unemployed or on unpaid leave (Table 6). In addition, residing in an inner regional area, higher capacity to save money and being retired or unemployed were also independent, statistically significant predictors of better health in the EQ-VAS multivariate regression analysis (Table 6). Sex was not a predictor of better health in the disutility models nor the EQ-VAS models (Table 6).

**Table 6:** Multivariate Quasipoisson regression analyses of EQ-5D-5L disutility scores, EQ-VAS scores, and sociodemographic variables

Variable	Disutility score			Disutility score			EQ-VAS score			EQ-VAS score		
	Model 1			Model 2			Model 1			Model 2		
	IRR	95% CI	p	IRR	95% CI	p	IRR	95% CI	p	IRR	95% CI	p
Control variables												
Intercept	**0.19**	**0.17–0.20**	** < 0.01**	**0.28**	**0.25–0.32**	** < 0.01**	**70.88**	**69.43–72.35**	** < 0.01**	**63.21**	**61.33**–**65.14**	** < 0.01**
Age category												
18–24 years^a^												
25–34 years	**0.86**	**0.78–0.95**	** < 0.01**	**0.84**	**0.77–0.93**	** < 0.01**	**1.03**	**1.00–1.05**	** < 0.05**	**1.03**	**1.01**–**1.06**	** < 0.05**
35–44 years	**0.70**	**0.63–0.78**	** < 0.01**	**0.68**	**0.61–0.75**	** < 0.01**	1.02	1.00**–**1.05	0.09	**1.03**	**1.00**–**1.06**	** < 0.05**
45–54 years	**0.75**	**0.67–0.83**	** < 0.01**	**0.72**	**0.66–0.80**	** < 0.01**	1.01	0.99–1.04	0.28	1.02	0.99–1.04	0.21
55–64 years	**0.68**	**0.61–0.75**	** < 0.01**	**0.59**	**0.53–0.66**	** < 0.01**	**1.05**	**1.02**–**1.08**	** < 0.01**	**1.05**	**1.03**–**1.08**	** < 0.01**
65–74 years	**0.68**	**0.60–0.76**	** < 0.01**	**0.51**	**0.44–0.59**	** < 0.01**	**1.07**	**1.04**–**1.09**	** < 0.01**	**1.08**	**1.05**–**1.12**	** < 0.01**
75 + years	**0.79**	**0.70–0.89**	** < 0.01**	**0.58**	**0.50–0.67**	** < 0.01**	**1.03**	**1.00**–**1.06**	** < 0.05**	**1.05**	**1.02**–**1.09**	** < 0.01**
Gender												
Male^a^												
Female	0.96	0.90– 1.01	0.13	0.99	0.94–1.04	0.66	1.01	1.00–1.02	0.10	1.00	0.99–1.02	0.52
Exploratory variables												
Remoteness area												
Major cities^a^												
Inner regional				**1.22**	**1.14**–**1.31**	** < 0.01**				**0.97**	**0.95**–**0.98**	** < 0.01**
Outer regional				**1.23**	**1.10**–**1.38**	** < 0.01**				0.99	0.96–1.01	0.32
Remote				1.32	0.84–1.96	0.20				1.04	0.93–1.15	0.53
Very remote				0.57	0.03–2.55	0.58				1.03	0.77–1.36	0.82
Finance												
I/We spend more money than I/we get^a^												
I/We have just enough money to get me/us through to the next pay day				**0.72**	**0.67**–**0.78**	** < 0.01**				**1.08**	**1.06**–**1.11**	** < 0.01**
There is some money left over each week but I/we just spend it				**0.61**	**0.54**–**0.68**	** < 0.01**				**1.11**	**1.08**–**1.15**	** < 0.01**
I/We can save a bit every now and then				**0.46**	**0.43**–**0.50**	** < 0.01**				**1.18**	**1.16**–**1.21**	** < 0.01**
I/We can save a lot				**0.34**	**0.31**–**0.38**	** < 0.01**				**1.25**	**1.22**–**1.28**	** < 0.01**
Employment												
Full time^a^												
Part time				1.07	0.98–1.16	0.15				1.00	0.98–1.02	0.73
Casual				1.01	0.89–1.15	0.82				1.00	0.97–1.03	0.90
Self-employed				**1.14**	**1.00**–**1.29**	** < 0.05**				1.00	0.97–1.03	0.88
Retired				**1.68**	**1.50**–**1.89**	** < 0.01**				**0.95**	**0.92**–**0.97**	** < 0.01**
Unemployed				**1.30**	**1.19**–**1.42**	** < 0.01**				**0.93**	**0.91**–**0.95**	** < 0.01**
On paid leave				1.01	0.70–1.40	0.96				0.98	0.91–1.06	0.60
On unpaid leave				1.35	0.98–1.80	0.05				0.94	0.86–1.02	0.14
QAIC	4267824^b^			4890353^b^			19609			19839		
QAICC	4272240^b^			4903870^b^			19629			19893		

Bold indicates the significant values where *p* < 0.05

*IRR* Incidence rate ratio, *QAIC* Quasi Akaike’s Information Criterion, *QAICC* Quasi Akaike’s Information Criterion—corrected, *EQ-VAS* EuroQol-Visual Analogue Scale

^a^Reference variable

^b^Calculated using disutility score*100 to remove decimal places

## Discussion

This study is the first to construct population norms for the EQ-5D-5L in a large nationally representative sample in Australia. It is also the first to compare HRQoL in Australia between sociodemographic variables. The results of this study can be used to supplement existing data, compare specific population groups with the general population, and inform healthcare research and policy making.

This study identified an EQ-5D-5L utility score of 0.86 and an EQ-VAS score of 73.2 in the general Australian population. This was lower than some of the previously reported national utility scores, such as those reported by China (0.96) [38], Japan (0.96) [20], Italy (0.93) [19], Vietnam (0.91) [24] and Poland (0.89) [21]. Countries with similar utility scores included New Zealand (0.85) [34], Canada (0.82) [39], Slovenia (0.81) [40], Norway (0.81) [41] and Iran (0.79) [42]. While the Australian utility scores were similar to comparable countries, such as New Zealand and Canada, the total and state utility scores were lower than the previously reported utility score in South Australia in 2016, of 0.91 [25]. These differences can be explained in part by the 2019 novel coronavirus pandemic (COVID-19). This study was conducted between July and August 2021, which was 16 months after the pandemic was declared by the World Health Organisation in March 11, 2020 [43]. The impact of COVID-19 on HRQoL has been observed in Sweden, which saw a reduction of 0.06 in EQ-VAS from pre-pandemic measurement in February 2020 to April 2020 and a reduction of 0.07 points between February 2020 and January 2021 [44]. The reduction in HRQoL was highest among the Swedish working age population [44]. This may indicate a shift, and probable reduction in national and global HRQoL.

This study found that HRQoL increased with age category. The opposite trend has been reported in many settings, with HRQoL (utility scores) generally decreasing as age increases, such as in Vietnam [24], Italy [19], Portugal [33], South Australia [25] and Poland [21]. As the data were weighted to the Australian population, this is unlikely to be due to a sample error. Although this was not measured directly, this unique finding may be attributed to the COVID-19 pandemic. This finding may also suggest that younger people were more affected by the physical and subsequent social isolation required to control the pandemic than older people. Recent literature supports this hypothesis with an Austrian study reporting a larger reduction in HRQoL across all domains (environmental, physical and social) in younger people when compared to older people post pandemic [45]. The increase in mental health issues reported in young people since the pandemic may also be contributing to the overall decline in HRQoL reported in younger people in other studies [46, 47].

The current study identified a higher percentage of young people (aged 18 to 24 years old) reporting a problem with anxiety/depression (72.3%) when compared to older people (aged 75 years or older) (35.5%). While higher levels of anxiety/depression in the youngest age groups have also been reported in Slovenia (52.0%)[40], Norway (43.2%)[41], Indonesia (40.1%)[48], China (32.1%)[49], and Hong Kong (30.1%)[50], they were considerably lower than this study. A previous population study conducted in South Australia in 2016 found the opposite trend, with 22.5% of people aged between 18 and 24 reporting a problem with anxiety/depression compared to 27.0% of people aged over 75 years old. By population, South Australia is the oldest jurisdictional population in the nation with a predominantly urban population and high levels of education, a combination of which may also contribute to the differences seen. A study from Poland found the same trend as the South Australian study [21]. This higher percentage of young people reporting a problem with anxiety/depression is also likely to be attributed to the COVID-19 pandemic [46]. A systematic review of the mental health changes of young people before and during the COVID-19 pandemic found that most studies reported a deterioration in the mental health in young people, with increased depression, anxiety and psychological distress after the pandemic started [47]. A Swedish study reported that during the pandemic, older people had significantly higher HRQoL when compared to younger age groups, when previously HRQoL had been consistent across all age categories [44]. This may indicate that younger people were impacted the most by the pandemic, and require more support, especially psychological support to return to their pre-COVID levels of HRQoL.

HRQoL tended to decrease as rurality increased in Australia. While this finding is challenging to compare to other countries—due to the differences in defining urban, regional, and rural populations—in general, this finding is consistent with previously reported literature. A recent study from China reported higher HRQoL in urban populations when compared to rural populations [38]. The same was found in Ireland, with people residing in urban areas experiencing better HRQoL when compared to people residing in rural areas [51], except for the dimension of anxiety and depression where people residing in urban areas exhibited greater anxiety and depression compared to people residing in rural areas [51]. In contrast, a study from 2020 in Quebec found no difference in HRQoL between their urban or rural populations [39]. Some of the reasons for the lower HRQoL and health in rural populations in Australia could be attributed to lower income, higher rates of alcohol use, occupational and physical risk taking as well as barriers to accessing health care, due to geographic spread, low population density, limited infrastructure, and the higher costs of delivering rural and remote health and social care [52]. This area continues to be a global challenge and a priority for many departments of health, including the Australian Government [53].

The largest strength of the current study is its large sample, which is a close representation of the Australian population and stratified by sex, age group and geographical region. However, the sample suffers from an under-representation of participants residing in the Northern Territory, which accounts for 0.9% of the Australian population [54]. Regardless of this issue, the current sample facilitated an exploration of the sociodemographic variations in HRQoL in Australia.

This study has several limitations. Firstly is the possibility of selection bias when conducting online surveys. Although the sample is generally representative for the Australian population in terms of key demographics (age, sex, state/territory of residence, rurality) it may be skewed towards participants with good computer literacy skills, especially as participants were registered with Qualtrics. This may have inflated the utility and EQ-VAS scores, as these are known to increase with education level [39, 50]. Secondly, potential social desirability bias may arise in studies involving the self-reporting of variables such as income. However, the use of an online survey may also mitigate social desirability bias in the EQ-5D responses, when compared face-to-face interviews. Thirdly, educational status was not collected nor included in the quotas or analyses. Therefore, the impact of the educational level of the participants on their HRQoL in the sample was unknown and was unable to be adjusted for. Fourthly, this study used cross-sectional data which provides a snapshot of the Australian general population at a certain point in time, thus, restricting the investigation of causal relationships between HRQoL and other sociodemographic characteristics over time. Finally, a potential limitation of this study is the time period in which data were collected, during the COVID-19 pandemic. This likely resulted in lower utility and EQ-VAS scores that may have been reported in a pre-COVID study. However, this may also assist researchers studying the impact of outbreaks such as COVID-19 on HRQoL to compare their findings with the Australian general population. Future research can also use the findings from this study to supplement existing data, compare specific population groups with the Australian general population, and inform healthcare research and policy making.

## Conclusion

This study is the first to construct population norms for the EQ-5D-5L in a large nationally representative sample in Australia. The utility scores in Australia were similar to previously reported utility scores in comparable countries. A major finding of this study was lower HRQoL in younger than older people, especially in the anxiety and depression domain. This may reflect a greater impact of the COVID-19 pandemic on the mental health of younger than older people, a finding that has been reported in other studies. This study can serve as a national baseline for HRQoL in Australia. These population norms will facilitate empirical HRQoL comparisons between the general population and more specific patient groups, and longitudinal studies of general HRQoL population surveys. Public health authorities and researchers may use the sociodemographic findings as a basis to further investigate the healthcare needs of the Australian population, such the high level of anxiety and/or depression reported in young people, and rural variations.

### Supplementary Information

Below is the link to the electronic supplementary material.

Supplementary file1 (DOCX 30 KB)

## References

[1] World Health Organization (WHO). (1946). *Preamble to the Constitution of the World Health Organization as adopted by the International Health conference*. New York.

[2] Callahan D (1973). The WHO definition of “health”. Stud Hastings Cent.

[3] Fayers P, Machin D (2007). Quality of life: The assessment, analysis and interpretation of patien-reported outcomes.

[4] Ravens-sieberer U (1994). Measuring and monitoring quality-of-life in population surveys : Still a challenge for public health research. Sozial- und Preventivmedizin SPM (Orley).

[5] Mbau R, Musiega A, Nyawira L, Tsofa B, Mulwa A, Molyneux S (2023). Analysing the efficiency of health systems: A systematic review of the literature. Applied Health Economics and Health Policy.

[6] Brazier J, Ratcliffe J, Saloman J, Tsuchiya A (2016). Measuring and valuing health benefits for economic evaluation.

[7] Yang F, Devlin N, Luo N (2019). ScienceDirect themed section: Evolution of EuroQoL cost-utility analysis using EQ-5D-5L data: Does how the utilities are derived matter ?. Value in Health.

[8] Centers for Disease Control and Prevention. (n.d.). Health-Related Quality of Life (HRQOL): Surveillance and Data. Retrieved April 6, 2023, from https://www.cdc.gov/hrqol/surveillance.htm

[9] Devlin NJ, Brooks R (2017). EQ-5D and the EuroQol Group: Past, present and future. Applied Health Economics and Health Policy.

[10] Herdman M, Gudex C, Lloyd A, Janssen M, Kind P, Parkin D, Badia X (2011). Development and preliminary testing of the new five-level version of EQ-5D (EQ-5D-5L). Quality of Life Research.

[11] Feng Y, Devlin N, Herdman M (2015). Assessing the health of the general population in England: How do the three- and five-level versions of EQ-5D compare ?. Health and Quality of Life Outcomes.

[12] Simone A, Christiansen J, Louise M, Møller S, Kronborg C, Jørgen K, Zöga S (2021). Comparison of the three—level and the five—level versions of the EQ—5D. The European Journal of Health Economics.

[13] EuroQol Research Foundation. (2023). EQ-5D-5L | Valuation. Retrieved September 1, 2023, from https://euroqol.org/eq-5d-instruments/eq-5d-5l-about/valuation-standard-value-sets/

[14] Devlin N, Parkin D, Janssen B (2020). Methods for analysing and reporting EQ-5D data.

[15] Jönsson B (1996). Quality of life and health economics: Where is the link?. Scandinavian Journal of Gastroenterology. Supplement.

[16] Tran BX, Hwang J, Nguyen LH, Nguyen AT, Reed N, Latkin K, Latkin CA (2016). Impact of socioeconomic inequality on access, adherence, and outcomes of antiretroviral treatment services for people living with HIV/AIDS in Vietnam. PLoS ONE.

[17] Hinz A, Kohlmann T (2014). The quality of life questionnaire EQ-5D-5L: Psychometric properties and normative values for the general German population. Quality of Life Research.

[18] Scalone L, Cortesi PA, Ciampichini R, Cesana G (2015). Health related quality of life norm data of the general population in Italy : Results using the EQ-5D-3L and EQ-5D-5L instruments. Epidemiology, Biostatistics, and Public Health.

[19] Meregaglia M, Malandrini F, Paolo A, Ciani O, Jommi C (2023). EQ-5D-5L population norms for Italy. Applied Health Economics and Health Policy.

[20] Shiroiwa T, Fukuda T, Ikeda S, Igarashi A, Noto S, Saito S, Shimozuma K (2016). Japanese population norms for preference-based measures: EQ-5D-3L, EQ-5D-5L, and SF-6D. Quality of Life Research.

[21] Golicki D, Niewada M (2017). EQ-5D-5L polish population norms. Archives of Medical Science.

[22] Olivares JCAPR (2016). Normative values of EQ-5D-5L: In a Spanish representative population sample from Spanish Health Survey, 2011. Quality of Life Research.

[23] Augustovski F, Irazola LRV, Morales M, Gibbons L, Manuel J, Ramos-gon JM (2016). An EQ-5D-5L value set based on Uruguayan population preferences. Quality of Life Research.

[24] Nguyen LH, Tran BX, Hoang LE, Tran TT, Latkin CA (2017). Quality of life profile of general Vietnamese population using EQ-5D-5L. Health and Quality of Life Outcomes.

[25] McCaffrey N, Kaambwa B, Currow DC, Ratcliffe J (2016). Health-related quality of life measured using the EQ-5D-5L: South Australian population norms. Health and Quality of Life Outcomes.

[26] Norman R, Mulhern B, Lancsar E, Lorgelly P, Ratcliffe J, Street D, Viney R (2023). The use of a discrete choice experiment including both duration and dead for the development of an EQ-5D-5L value set for Australia. PharmacoEconomics.

[27] Australian Bureau of Statistics (ABS). (2016). 2016 Census QuickStats: Australian Bureau of Statistics. Retrieved April 6, 2023, from https://www.abs.gov.au/census/find-census-data/quickstats/2016/0

[28] Kochovska S, Chang S, Ferreira D, Brunelli VN, Luckett T, Morgan L, Currow DC (2022). Invisibility of breathlessness in clinical consultations: A cross-sectional, national online survey. European Respiratory Journal.

[29] Australian Bureau of Statistics (ABS). (n.d.). Australian Statistical Geography Standard (ASGS). Retrieved February 10, 2023, from www.abs.gov.au/statistics/statistical-geography/australian-statistical-geography-standard-asgs

[30] Devlin NJ, Shah KK, Feng Y, Mulhern B, van Hout B (2018). Valuing health-related quality of life: An EQ-5D-5L value set for England. Health Economics (United Kingdom).

[31] Group TE (1990). EuroQol—A new facility for the measurement of health-related quality of life. Health Policy.

[32] Australian Bureau of Statistics (ABS). (2021). Snapshot of Australia. *ABS*. Retrieved February 21, 2023, from https://www.abs.gov.au/statistics/people/people-and-communities/snapshot-australia/latest-release.

[33] Ferreira PL, Pereira LN, Antunes P, Ferreira LN (2023). EQ-5D-5L Portuguese population norms. European Journal of Health Economics.

[34] Sullivan T, Turner RM, Derrett S, Hansen P (2021). New Zealand population norms for the EQ-5D-5L constructed from the personal value sets of participants in a national survey. Value in Health.

[35] Corp IBM (2013). IBM SPSS statistics for windows, version 22.0.

[36] RStudio Team. (2020). RStudio: Integrated Development for R. Boston, MA: PBC. Retrieved from http://www.rstudio.com/.24

[37] Vandenbroucke JP, Von Elm E, Altman DG, Gøtzsche PC, Mulrow CD, Pocock SJ, Egger M (2007). Strengthening the reporting of observational studies in epidemiology (STROBE): Explanation and elaboration. PLoS Medicine.

[38] Liao M, Luo N, Rand K, Yang Z (2023). Urban/rural differences in preferences for EQ-5D-5L health states: A study of a multi-ethnic region in China. Quality of Life Research.

[39] Poder TG, Carrier N, Kouakou CRC (2020). Quebec health-related quality-of-life population norms using the EQ-5D-5L: decomposition by sociodemographic data and health problems. Value in Health.

[40] Rupel VP, Ogorevc M (2020). EQ-5D-5L Slovenian population norms. Health and Quality of Life Outcomes.

[41] Garratt AM, Hansen TM, Augestad LA, Rand K, Stavem K (2022). Norwegian population norms for the EQ-5D-5L: Results from a general population survey. Quality of Life Research.

[42] Emrani Z, Sari AA, Zeraati H, Olyaeemanesh A, Daroudi R (2020). Health-related quality of life measured using the EQ-5D—5 L: Population norms for the capital of Iran. Health and Quality of Life Outcomes.

[43] World Health Organization (WHO). (2020). WHO Director-General’s opening remarks at the media briefing on COVID-19 - 11 March 2020. *WHO*. Retrieved April 7, 2023, from https://www.who.int/director-general/speeches/detail/who-director-general-s-opening-remarks-at-the-media-briefing-on-covid-19---11-march-2020

[44] Persson U, Olofsson S, Gu NY, Gong CL, Jiao X, Hay JW (2021). Quality of life in the Swedish general population during COVID-19—Based on pre- and post-pandemic outbreak measurement. Nordic Journal of Health Economics.

[45] Dale R, Budimir S, Probst T, Humer E, Pieh C (2022). Quality of life during the COVID-19 pandemic in Austria. Frontiers in Psychology.

[46] Xiong J, Lipsitz O, Nasri F, Lui LMW, Gill H, Phan L, McIntyre RS (2020). Impact of COVID-19 pandemic on mental health in the general population: A systematic review. Journal of Affective Disorders.

[47] Kauhanen L, Mohd W, Wan A, Yunus M, Lempinen L, Peltonen K, Sourander A (2022). A systematic review of the mental health changes of children and young people before and during the COVID—19 pandemic. European Child & Adolescent Psychiatry.

[48] Purba FD, Hunfeld JAM, Iskandarsyah A, Fitriana S, Sadarjoen SS, Passchier J, Busschbach JJV (2018). Quality of life of the Indonesian general population : Test-retest reliability and population norms of the EQ-5D-5L and WHOQOL-BREF. PLoS ONE.

[49] Yang Z, Busschbach J, Liu G, Luo N (2018). EQ-5D-5L norms for the urban Chinese population in China. Health and Quality of Life Outcomes.

[50] Wong EL, Cheung AW, Wong AY, Xu RH, Ramos-goñi JM, Rivero-arias O (2019). Normative profile of health-related quality of life for Hong Kong general population using preference-based instrument EQ-5D-5L. Value in Health.

[51] Hobbins A, Barry L, Kelleher D, Neill CO (2018). The health of the residents of Ireland: Population norms for Ireland based on the EQ-5D-5L descriptive system—A cross sectional study. HRB Open Research.

[52] Australian Institute of Health and Welfare. (n.d.). Rural and remote health. Retrieved April 5, 2023, from https://www.aihw.gov.au/reports/rural-remote-australians/rural-and-remote-health#Profile of rural and remote Australians

[53] National Rural Health Alliance. (2022). Australian election 2022 rural health priorities. Retrieved from https://www.ruralhealth.org.au/sites/default/files/documents/NRHA_Aust_election_2022_rural_health_priorities.pdf

[54] Australian Bureau of Statistics (ABS). (2021). Australia 2021 Census all persons QuickStats. *ABS*. Retrieved November 21, 2022, from https://www.abs.gov.au/census/find-census-data/quickstats/2021/AUS

