# Supplementary material for: Australian population norms for health-related quality of life measured using the EQ-5D–5L, and relationships with sociodemographic characteristics
Source: Qual Life Res. 2023 Dec 12;33(3):721–33. doi: 10.1007/s11136-023-03558-z (PMC10894099; doi:10.1007/s11136-023-03558-z)
Supplement: Supplementary file 1 — Supplementary file1 (DOCX 30 KB) [file 11136_2023_3558_MOESM1_ESM.docx]

**Supplementary materials:**

**Sensitivity analysis: Comparing a US**[1] **and Italian**[2] **value set, developed using the** **EQ-VT v2.1 protocol, with an Australian value set** [3]**, developed using the** **DCE duration valuation protocol, on mean utility scores across age and sex in an Australian representative sample.**

The mean utility score calculated using the Australian value set was higher when compared to the mean calculated using the US and Italian value set (mean=0.85 vs 0.77 and 0.80 respectively). The mean utility scores from the Australian value set were higher across all sex and age categories. The mean utility scores from the US and Italian value sets were lower than the Australian value set, however they followed the same trends (supplementary Figure 1).

## Supplementary Table 1: Mean EQ-5D-5L utility scores using the Australian, US and Italian value set by age and sex

|  |  | Male | | | Female | | | Total | | |
| --- | --- | --- | --- | --- | --- | --- | --- | --- | --- | --- |
| Variable | | Australia | US | Italy | Australia | US | Italy | Australia | US | Italy |
| Age category, years | | 0.85 | 0.72 | 0.78 | 0.74 | 0.62 | 0.65 | 0.79 | 0.66 | 0.71 |
|  | 18-24 | 0.83 | 0.73 | 0.77 | 0.82 | 0.71 | 0.75 | 0.82 | 0.71 | 0.75 |
|  | 25-34 | 0.83 | 0.74 | 0.78 | 0.85 | 0.76 | 0.80 | 0.84 | 0.75 | 0.79 |
|  | 35-44 | 0.87 | 0.79 | 0.83 | 0.86 | 0.77 | 0.81 | 0.87 | 0.78 | 0.82 |
|  | 45-54 | 0.86 | 0.77 | 0.81 | 0.85 | 0.75 | 0.79 | 0.85 | 0.76 | 0.80 |
|  | 55-64 | 0.85 | 0.77 | 0.81 | 0.87 | 0.79 | 0.82 | 0.86 | 0.77 | 0.81 |
|  | 65-74 | 0.89 | 0.82 | 0.86 | 0.87 | 0.79 | 0.83 | 0.88 | 0.81 | 0.85 |
|  | 75+ | 0.90 | 0.82 | 0.86 | 0.84 | 0.73 | 0.79 | 0.88 | 0.79 | 0.84 |
|  | Total | 0.86 | 0.78 | 0.82 | 0.85 | 0.75 | 0.79 | 0.85 | 0.77 | 0.80 |

US = United States

**Supplementary Figure 1: Comparing the Australian, US and Italian EQ-5D-5L value sets on the utility scores across age and sex in an Australian representative sample**

References:

1. Pickard, A. S., Law, E. H., Jiang, R., Pullenayegum, E., Shaw, J. W., Xie, F., … Busschbach, J. J. V. (2019). United States Valuation of EQ-5D-5L Health States Using an International Protocol. *Value in Health*, *22*(8), 931–941. https://doi.org/10.1016/j.jval.2019.02.009

2. Finch, A. P., Meregaglia, M., Ciani, O., Roudijk, B., & Jommi, C. (2022). An EQ-5D-5L value set for Italy using videoconferencing interviews and feasibility of a new mode of administration. *Social Science and Medicine*, *292*, 114519. https://doi.org/10.1016/j.socscimed.2021.114519

3. Norman, R., Mulhern, B., Lancsar, E., Lorgelly, P., Ratcliffe, J., Street, D., & Viney, R. (2023). The Use of a Discrete Choice Experiment Including Both Duration and Dead for the Development of an EQ-5D-5L Value Set for Australia. *PharmacoEconomics*, *41*(4), 427–438. https://doi.org/10.1007/s40273-023-01243-0
